# Molecular Mechanisms of *MYCN* Dysregulation in Cancers

**DOI:** 10.3389/fonc.2020.625332

**Published:** 2021-02-03

**Authors:** Ruochen Liu, Pengfei Shi, Zhongze Wang, Chaoyu Yuan, Hongjuan Cui

**Affiliations:** ^1^ State Key Laboratory of Silkworm Genome Biology, College of Sericulture, Textile and Biomass Sciences, Southwest University, Chongqing, China; ^2^ Cancer Center, Reproductive Medicine Center, Medical Research Institute, Southwest University, Chongqing, China; ^3^ NHC Key Laboratory of Birth Defects and Reproductive Health (Chongqing Key Laboratory of Birth Defects and Reproductive Health, Chongqing Population and Family Planning Science and Technology Research Institute), Chongqing, China

**Keywords:** MYCN, cancer, gene amplification, G-quadruplex, NCYM, super enhancer, synthetic lethality

## Abstract

*MYCN*, a member of *MYC* proto-oncogene family, encodes a basic helix-loop-helix transcription factor N-MYC. Abnormal expression of N-MYC is correlated with high-risk cancers and poor prognosis. Initially identified as an amplified oncogene in neuroblastoma in 1983, the oncogenic effect of N-MYC is expanded to multiple neuronal and nonneuronal tumors. Direct targeting N-MYC remains challenge due to its “undruggable” features. Therefore, alternative therapeutic approaches for targeting *MYCN*-driven tumors have been focused on the disruption of transcription, translation, protein stability as well as synthetic lethality of *MYCN*. In this review, we summarize the latest advances in understanding the molecular mechanisms of *MYCN* dysregulation in cancers.

## Introduction

**Figure 1:**
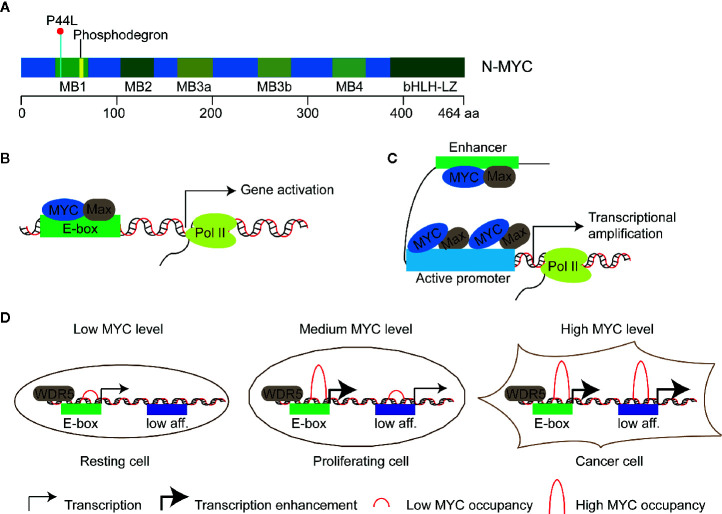
Models of transcriptional regulation of target genes by MYC proteins. **(A)** Schematic diagram of N-MYC protein structure. Five highly conserved stretched called MYC boxes 1 to 4 (MB) and the basic helix-loop-helix-leucine-zipper (bHLH-LZ) domain at the C terminus are shown. The recurrent somatic mutation P44L and the putative N-MYC phosphodegron are shown in cyanine and yellow respectively. **(B)** Gene-specific regulation model: MYC/Max dimer binds and regulates a subset of genes with E-boxes in their promoters. **(C)** Global gene activation model: MYC accumulates in the promoter regions of active genes independent of E-box and leads to transcriptional amplification in cancer cells with high level of MYC proteins (4). **(D)** Gene-specific affinity model: high-affinity binding sites, such as those with E-boxes and WDR5 (WD-repeat protein 5) binding, are already fully occupied by MYC at physiological MYC protein level (medium level) in proliferating cells; low-affinity (low aff.) binding sites can be occupied by MYC at oncogenic MYC protein level (high level) in cancer cells (5).

N-MYC is a transcription factor of the MYC oncogene family. This gene family of humans consists of three members, namely, *MYCC*, *MYCN*, *MYCL*, which encodes C-MYC, N-MYC, and L-MYC protein respectively (“MYC” was used to indicate all three genes in this review). The first identified *MYC* gene was *MYCC* as a homolog of an avian retroviral gene *v-myc*, then *MYCN* in neuroblastoma and *MYCL* in lung cancer (1–3). These proteins show similar structure with the highest homology in five short stretches called MYC boxes 1 to 4 at the N terminus and in the basic helix-loop-helix-leucine-zipper (bHLH-LZ) domain at the C terminus (**Figure 1A**) (6–9). The former enables MYC to interact with different effector proteins including TRRAP and P400 which mediate chromatin remodeling and modification (10, 11), the latter allows MYC to form a heterodimer with partner proteins that also contain a bHLH-LZ domain, such as MAX. MYC/MAX heterodimer bind to the target motif called E-box with the consensus sequence of CAC(G/A)TG to regulate the expression of targeted genes (**Figure 1B**). In addition, MYC can also bind to targeted sequences that show deviation from or no similarity to the E-box, suggesting the association of MYC to chromatin can be instructed by other factors (12, 13). For example, MYC can invade promoter regions of active genes and cause global transcriptional amplification (**Figure 1C**) (4, 14, 15). The two different action modes of MYC seem conflicting, *i.e.*, gene-specific regulation model versus global gene activation model. The third model, gene-specific affinity model, in which the affinity of promoters for MYC is different and relies on the MYC levels and the interaction of MYC with core promoter-binding factors, such as WDR5 (**Figure 1D**), has been proposed to reconcile the action modes of MYC (5, 16). MYC proteins affect transcription of a large number of genes and thus regulate fundamental cellular processes, including proliferation, metabolism, apoptosis, differentiation, and immune surveillance (17–21).

**Figure 2:**
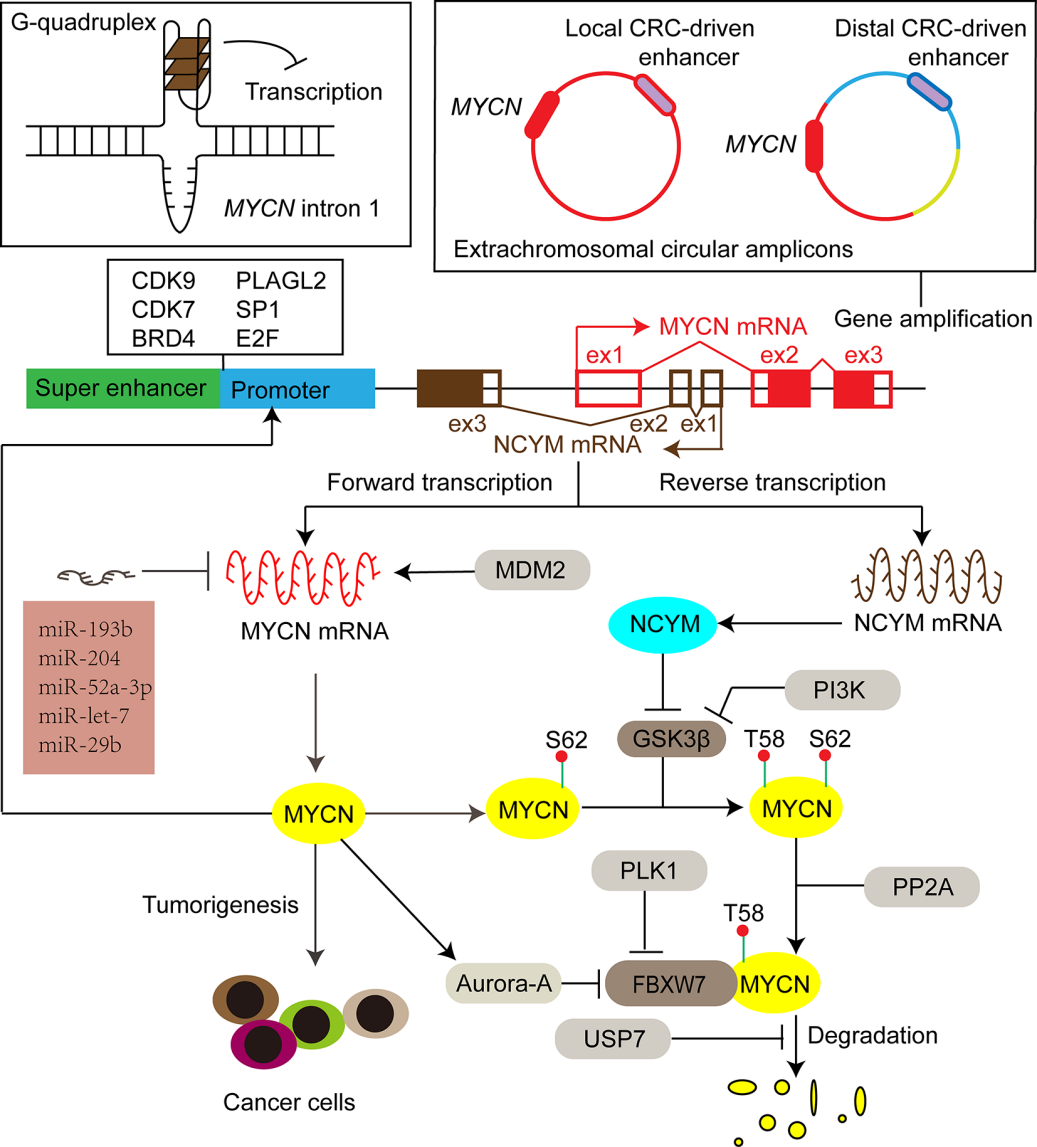
The expression of *MYCN* is activated or repressed at DNA, mRNA and protein levels by different factors, including secondary DNA structure, enhancers, transcription factors, miRNAs, ubiquitination-dependent proteasome degradation machinery and its cis-antisense gene *NCYM*. Filled red and brown boxes indicate translated regions of MYCN and NCYM respectively, while the blank counterparts represent untranslated regions. CRC core regulatory circuitry, CDK9 cyclin-dependent kinase 9, CDK7 cyclin-dependent kinase 7, BRD4 bromodomain-containing 4, PLAGL2 pleiomorphic adenoma gene-like 2, SP1 specific protein 1, GSK3β glycogen synthase kinase 3β, PI3K phosphoinositide 3-kinase, FBXW7 F-box and WD repeat domain-containing 7, PP2A protein phosphatase 2A, PLK1 polo-like kinase 1, USP7 ubiquitin-specific protease 7, MDM2 murine double minute 2.

With evolutionarily conserved domains, the three MYC proteins share certain extent of functional redundancy. For instance, when N-MYC is expressed from the *MYCC* locus, it can rescue development, cellular growth, and differentiation in *MYCC* deficient mice (22). On the other hand, C-MYC, N-MYC, and L-MYC have their own unique features. Enhanced expression of different MYC paralogs induces tumors with different biological characteristics in medulloblastoma (23, 24), prostate cancer (25), and lung cancer (26). Furthermore, the amplification of *MYC* genes is mutually exclusive, and the switch of gene expression among the members is associated with cell lineage shift, tumor progression, and treatment resistance (27, 28). Different collaborative proteins of MYC paralogs help to demarcate a unique subset of responsive genes, which could partially explain the distinct biological functions among MYC members. For example, N-MYC interacts with TWIST1 at enhancers to activate developmental genes important to neuroblastoma tumorigenesis, while TCF3 (E2A) is selectively required for progression of C-MYC driven myeloma (15). In this mini-review, we focus on N-MYC-driven tumors. Since discovered in 1983 in neuroblastoma (1, 3), the oncogenic effect of N-MYC has been demonstrated both in various neuronal [*e.g.*, glioblastoma (29), medulloblastoma (30), astrocytoma (31)], and nonneuronal [*e.g.*, prostate cancers (32), breast cancers (33), hematologic malignancies (34), pancreatic tumors (35), Wilms tumors (36), hepatocellular carcinoma (37), rhabdomyosarcoma (38), ovarian cancers (39)] tumors. Specifically, this mini-review summarizes the latest advances in the regulation network of N-MYC expression (**Figure 2**) and the related therapeutic targets for *MYCN*-driven tumors.

## Molecular Mechanisms of *MYCN* Dysregulation and the Therapeutic Targets

The tissue specificity and strength of *MYC* gene expression are under tight control in normal circumstances. Studies of mice show that the expression of *MYCN* is high during early developmental stages and in specific tissues including forebrain, hindbrain, and kidney of newborn mice, while *MYCC* is broadly expressed throughout the tissues and the developmental stages analyzed. Clinical observation of *MYCN* amplification in human neuroblastoma firstly pointed out the potential association between *MYCN* gene and tumorigenesis (1, 3). Although amplified DNAs encompassing *MYCN* are more than 100 kb and can include adjacent co-amplified genes, *MYCN* has emerged as the only consistently amplified gene (40). Using transgenic animal models, multiple studies establish that N-MYC overexpression is a driver of cancers. For example, targeted expression of human N-MYC causes neuroblastoma in transgenic mice and zebrafish (41, 42). Neuroblastomas with enhanced expression of N-MYC without *MYCN* amplification are known to be similarly high-risk and poor prognosis (43). Recent studies show that high N-MYC protein and RNA levels could be better biomarkers than *MYCN* gene amplification in predicting the prognosis of neuroblastoma patients (44, 45), underscoring the importance of aberrant expression of N-MYC in tumor progression. Here, we discuss mechanisms of *MYCN* dysregulation at DNA, mRNA and protein levels, and corresponding therapeutic targets.

## Gene Amplification of *MYCN*

Gene amplification is a frequent mechanism that can cause proto-oncogene overexpression. It is a process that involves unscheduled DNA replication, recombination and/or formation of extrachromosomal DNA, leading to a selective increase of gene copy number up to several hundred (40). The occurrence of proto-oncogene amplification can be detected by the presence of “double minutes” or “homogeneously staining chromosomal regions”. *MYCN* was the first discovered paradigm of proto-oncogene amplification and is an important bio-marker to stratify clinical risk. It was initially detected in about 20% to 25% of neuroblastoma, then at a much lower incidence in small cell lung cancer, retinoblastoma, hepatocellular carcinoma, malignant gliomas, and peripheral neuroectodermal tumors (46, 47). Amplification of *MYCN* has been recognized as a consequence of genomic instability and occurs sporadically (48). Overexpression of N-MYC initiates tumorigenesis by preventing the normal physiological process of neural crest cell death in *TH-MYCN* transgenic mice in which human *MYCN* is under the control of a tyrosine hydroxylase (TH) promoter, and the formation of neuroblastoma involves further changes of the persisting embryonal neural crest cells, including *MYCN* amplification (49). In addition, *MYCN* amplification is associated with advanced neuroblastomas, suggesting that the amplification is a late event during the tumorigenesis (49–51)

Although multiple replication-based mechanisms, such as double rolling-circle replication, have been proposed to explain gene amplification, the important factors that induce and regulate *MYCN* amplification remain to be completely investigated (52–55). Proto-oncoprotein c-MYB transcription factor is implicated in the regulation of cell growth and proliferation of neuroblastoma (56). The functional ortholog of *Drosophila melanogaster*, Dm-Myb, is directly implicated in the site-specific DNA replication, leading to amplification of the chromosomal loci with the chorion gene cluster (57). Aygun and Altungoz showed that c-MYB is involved in the control of *MYCN* amplification in *MYCN*-amplified neuroblastoma cell lines (58). Specifically, the *MYCN* gene dosage is increased upon knockdown of c-MYB expression, which may be associated with the elevated expression of geminin protein that causes a shift from genomic DNA replication to *MYCN* amplification (58–60). Recent sequencing studies indicate that the structure of extrachromosomal *MYCN* amplicons are shaped by enhancer sequences (61, 62). Specifically, Helmsauer et al. reported two distinct classes of extrachromosomal circular *MYCN* amplicons: the first class co-amplifies a local core regulatory circuitry (CRC)-driven enhancer; the second class shows a complex chimeric structure with a distal CRC-driven enhancer instead of the local enhancer (**Figure 2**) (61). Long inverted repeats and microhomology are significantly associated with boundary regions of the *MYCN* amplicon units, and thus might also be involved in the initiation or regulation of *MYCN* amplification (55, 58). Elucidating the mechanisms of *MYCN* amplification may bring about new therapeutic strategies targeting *MYCN* amplification to treat *MYCN*-driven tumors.

Although the amplified genes tend to overexpress, gene amplification not necessarily leads to high level of gene expression. In fact, there is inconsistency between *MYCN* gene dosage, mRNA and protein levels, and clinical outcomes (44, 63). For example, low DNA dosage but high RNA level is detected in some neuroblastoma samples, while high DNA dosage but low RNA level in some other samples (45). Additional *MYCN* gene copies may also suppress their own expression (58). Genome-wide analysis in humans and some model organisms revealed that genes in copy number variation regions are expressed at lower and more variable levels than genes mapped elsewhere (64). Alternatively, as in plants, repeated genes may suffer from homology-dependent gene silencing that involves DNA methylation or histone modification (65, 66). Consistently, only a weak positive correlation of *MYCN* expression with copy number is detected in Wilms tumor, while a strong negative correlation of *MYCN* expression with DNA methylation level at specific loci is observed (67). Importantly, transcriptional and posttranscriptional regulation determines the final level of N-MYC protein in both *MYCN* amplified and non-amplified tumors. For instance, enhancer hijacking that repositions a super enhancer close to the affected genes through chromosomal translocation accounts for the high level of C-MYC or N-MYC expression in some neuroblastoma cells without *MYCC* amplification or without a high *MYCN* copy number, respectively (68, 69).

## Regulation of *MYCN* Transcription

### Super Enhancer and Transcription Factors

A general feature of *MYC* genes is their transcriptional regulation by upstream super enhancers (SEs) (70). SE regions are occupied by abundant transcription factors, cofactors, and chromatin regulators, thereby promoting transcription of *MYC* genes (71). Specifically, H3K27 acetylation (H3K27ac), a marker of active enhancers and promoters, is enriched in the SE regions and recognized by BRD4 of bromodomain and extra-terminal domain (BET) protein family that recruits positive transcription elongation factor b (P-TEFb) to the promoters to phosphorylate RNA polymerase II, and thus facilitates transcriptional initiation, pause release and elongation (72–74). BET inhibitors, such as JQ1 and OTX015, can displace the BRD4 oncoprotein from chromatin (75), which potently represses *MYCN* transcription in neuroblastoma cell lines and effectively reduces neuroblastoma cell viability *in vitro* and *in vivo* (76, 77). It has been reported that the toxic effects of BET inhibitors depend on p53 (78). The combination of MDM2 (an E3-ubiquitin ligase involved in proteasomal degradation of p53) inhibitor (CGM097) and OTX015 results in p53 activation and decreased expression of MYC proteins, which synergistically promotes neuroblastoma cell death (79). A recent study shows that triple-negative breast cancer (TNBC) cells with high expression of *MYCN* are also sensitive to BET inhibitors (80). Furthermore, combined BET and MEK inhibition synergistically represses the growth of *MYCN*-expressing patient-derived xenograft TNBC tumors (80).

Besides BET proteins, transcriptional cyclin-dependent kinases (CDKs) are recruited to SEs, especially CDK7, a catalytic subunit of the transcription factor IIH complex (TFIIH), and CDK9, a kinase subunit of P-TEFb (81, 82). These CDKs regulate the transcriptional cycle of RNA polymerase II *via* phosphorylating the C-terminal domain of the polymerase, which enhances expression of SE-associated oncogenes, such as *MYCN* (83–85). A covalent inhibitor of CDK7, THZ1, selectively targets *MYCN*-amplified neuroblastoma cells, leading to global suppression of *MYCN*-dependent transcriptional amplification and sustained growth inhibition of tumors in a mouse model of neuroblastoma (85). CYC065 (fadraciclib), a clinical inhibitor of CDK9 and CDK2 (a major regulator of apoptotic cell death), selectively targets *MYCN*-amplified neuroblastoma through a loss of *MYCN* transcription and growth arrest, followed by sensitizing cells for apoptosis as a result of CDK2 inhibition (86). Furthermore, the combined use of CYC065 with temozolomide (a reference therapy for relapsed neuroblastoma), leads to long-term repression of neuroblastoma growth *in vivo* (86).

Recent studies reveal that several super-enhancer harboring transcription factors including HAND2, ISL1, PHOX2B, GATA3, and TBX2 constitute a CRC that is essential for the *MYCN* expression and the survival of *MYCN*-amplified neuroblastoma cells (61, 87). BRD4 and CDK7 inhibitors synergistically repress the expression of all the CRC transcription factors and N-MYC, which inhibits neuroblastoma cell growth (87). Knockdown of each CRC transcription factors also suppresses the expression of *MYCN* (87). Interestingly, the CRC-driven enhancers (local or distal) are associated with extrachromosomal circular *MYCN* amplicons (**Figure 2**) (61), underscoring the role of the CRC transcription factors in the regulation of *MYCN* expression.

Other transcription factors, such as specific protein 1 (SP1) (88), E2F (89), and pleiomorphic adenoma gene-like 2 (PLAGL2) (90), participate in the regulation of *MYCN* expression. The three transcription factors directly bind to the cognate binding sites in the *MYCN* promoter, contributing to *MYCN* activation. Moreover, N-MYC regulates *PLAGL2* transcription through five N-MYC-binding E-boxes in the *PLAGL2* promoter region, forming a positively regulatory loop between the two transcription factors, which is crucial for expression of each other in neuroblastoma tumors (90). Lipid desaturation-associated endoplasmic reticulum (ER) stress inhibits *MYCN* expression *via* upregulating the transcriptional repressor ATF3 in hepatocellular carcinoma cells (91). Since these transcription factors including SP1, E2F2, and PLAGL2 are involved in the regulation of *MYCN* expression, they mediate the effects of metabolic change and pharmacological treatment on *MYCN* expression and *MYCN*-driven tumors (92). Aldehyde dehydrogenase family 18 member A1 (ALDH18A1) is a key enzyme for the synthesis of proline from glutamate and plays important role in the proliferation, self-renewal, and tumorigenicity of neuroblastoma cells (93). ALDH18A1 promotes the transcription of *MYCN via* the *miR-29b*/SP1 regulatory loop. ALDH18A1-specific inhibitor, YG1702, inhibits *MYCN* expression and attenuates the growth of human neuroblastoma (93). All-trans retinoic acids have been used for neuroblastoma therapy for decades by inhibiting the expression of *MYCN* and inducing the neuronal differentiation of neuroblastoma cells (94–96). Loss of E2F binding or suppression of PLAGL2 expression mediates the negative regulation of *MYCN* expression by retinoic acid (89, 90). Acyclic retinoid dampens *MYCN* gene expression and suppresses cell proliferation of *MYCN*-overexpressed hepatocellular carcinoma cells, at least in part by ER stress-induced ATF3 signaling pathway (91).

### G-Quadruplex

Another feature of *MYC* genes is their transcriptional regulation by non-B DNA structures including single-stranded bubbles, Z-DNA, and G-quadruplexes (97). G-quadruplexes are four-stranded DNA secondary structures and consist of stacked G-quartets that formed by the assembly of four Hoogsteen hydrogen-bonded guanines in guanine-rich regions of DNA. A G-quadruplex forming sequence lies in the promoter of *MYCC* gene (98) and in intron 1 of *MYCN* gene (99) respectively. This sequence exists in equilibrium between transcriptionally active forms (double helical and single stranded) and a silenced form (G-quadruplex), which controls up to 90% of *MYCC* transcription (100). Thus, targeting *MYC* expression through G-quadruplex stabilization becomes an attractive candidate for the treatment of *MYC*-driven tumors. Cationic porphyrin TMPyP4 is a small molecule able to stabilize G-quadruplex structure and efficiently repress *MYCC* transcription, which establishes the principle that MYC transcription can be controlled by ligand-mediated G-quadruplex stabilization (98). A cell penetrating thiazole peptide, TH3, shows improved targeting specificity to *MYCC* G-quadruplex over other tested G-quadruplexes (100). This peptide down-regulates *MYCC* expression in cancer cells and reduces proliferative activities by inducing S phase cell cycle arrest and apoptosis (100). Nucleolin is a protein involved in the folding the G-quadruplex (101). Quarfloxin (CX-3543), a fluoroquinolone-based antitumor agent, can inhibit *MYCC* expression by redistribution of nucleolin from the nucleolus to the nucleoplasm to bind to *MYCC* G-quadruplex (102). Treating neuroblastoma cells with quarfloxin represses N-MYC expression and causes G2-cell cycle arrest and apoptosis (103). The most profound anti-tumor effects of quarfloxin are associated with *MYCN* amplification (103), implying the above drugs that target *MYCC* G-quadruplex can also be used to target *MYCN* G-quadruplex for treatment of *MYCN*-driven tumors.

## Posttranscriptional Regulation of *MYCN* mRNA

Along with transcription factors, noncoding RNAs including long noncoding RNA (lncRNAs) and microRNAs (miRNAs) are involved in the regulatory network of *MYCN* expression. miR-506-3p is a potent differentiation inducer and a strong repressor of *MYCN* expression in neuroblastoma cells by targeting PLAGL2 transcription factor (90, 104). miR-204 directly binds *MYCN* mRNA, represses *MYCN* expression, and inhibits a subnetwork of oncogenes that strongly correlate with *MYCN*-amplified neuroblastoma and poor patient outcome (105). miR-193b targets several important oncogenes including *MYCN* and is expressed at low levels in neuroblastoma cell lines (106). *MYCN* mRNA is a direct target of miR-520c-3p in cholangiocarcinoma, and transcription factor SP1-induced lncRNA HOXD-AS1 enhances *MYCN* expression through competitively binding to miR-520c-3p, which associates with lymph node invasion, advanced TNM stage and poor prognosis (107). A miRNA network, consisting of miR-29b, miR-29a, and miR-193b, mediates posttranscriptional regulation of the *MYCN* expression by ALDH18A1 (93, 108). miRNA let-7 is a strong negative regulator of *MYCN* expression and can inhibit proliferation and clonogenic growth of *MYCN*-amplified neuroblastoma cells (108). LIN28B, an RNA-binding protein and a suppressor of microRNA biogenesis, selectively blocks the biogenesis of let-7 miRNA, consequently leading to increased *MYCN* expression in neuroblastoma cells (109). These results indicate that *MYCN* is targeted by several miRNAs. Increased expression of these miRNAs inhibits cell proliferation and tumorigenesis (105). Furthermore, miR-506-3p has been reported to mediate the antitumor effect of retinoic acid in neuroblastoma cells (90). These results underscore the potential of miRNA-based anticancer therapy. Interestingly, the E3-ubiquitin ligase MDM2 increases the *MYCN* mRNA stability and translation by binding to AU-rich elements of the 3′ UTR of *MYCN* mRNA through its C-terminal RING domain (110). RNAi-mediated knockdown of MDM2 leads to remarkable suppression of neuroblastoma cell growth and induction of cell death through a p53-independent pathway (110).

## Regulation of *MYCN* Translation

Efficient translation guarantees the oncogenic level of N-MYC protein. N-MYC has been shown to promote the expression of many genes involved in ribosome biogenesis and protein synthesis (111), suggesting N-MYC contributes to its own overexpression by enhancing the capacity of translation. The N-MYC protein level is decreased as a result of ribosome biogenesis inhibition (103). Mammalian target of rapamycin (mTOR) is a serine/threonine protein kinase that controls initiation of protein translation (112). mTOR directly phosphorylates and inactivates eukaryotic translation initiation factor 4E (eIF4E)-binding protein 1 (4E-BP1), which leads to activation of eIF4E and thus promotes cap-dependent translation of mRNAs including MYC family (112). Pharmacological inhibition of the AKT/mTOR pathway reduces N-MYC level and exhibits therapeutic efficacy in *MYCN*-amplified neuroblastoma (113, 114).

## Regulation of N-MYC Stability

After translation, the stability and activity of *N-MYC* protein are tightly controlled by ubiquitination-dependent proteasome degradation that is a brake in the *MYCN*-driven cancers. The degradation of the N-MYC proto-oncoprotein in neural stem/progenitor cells is required for the arrest of proliferation and the start of differentiation. Two E3 ubiquitin ligases FBXW7 and HUWE1 ubiquitinate N-MYC through Lys 48-mediated linkages and target it for destruction by the proteasome (115, 116). The recognition of N-MYC by FBXW7 involves several sequential reactions, *i.e.*, phosphorylation on Ser62 by CDK1 (117), phosphorylation on Thr58 by glycogen synthase kinase 3β (GSK3β), dephosphorylation of Ser62 by protein phosphatase 2A (PP2A) (118), which facilitates the Thr58 phosphorylated N-MYC binding with FBXW7 (116).

Dysregulation of the degradation process will cause the accumulation of N-MYC protein to the oncogenic level. Aurora-A, a member of the Aurora kinase family, is identified in an shRNA screen of genes that are highly expressed in *MYCN*-amplified neuroblastoma cells and contributes to the stabilization of N-MYC (119). Mechanistically, the catalytic domain of Aurora-A interacts directly with N-MYC through binding sites that flank either side of MYC box 1 which contains the phosphodegron (Thr58) recognized by FBXW7, thereby preventing the binding of FBXW7 with N-MYC substrate (120). Furthermore, the expression of Aurora-A is increased in the *MYCN*-amplified neuroblastoma, suggesting a potential feed-forward loop that improves the stability of both proteins (121). Two Aurora-A kinase activity inhibitors, MLN8054 and MLN8237, disrupt the Aurora-A/N-MYC complex and promote FBXW7-mediated degradation of N-MYC, which correlates with tumor regression and prolonged survival in a mouse model of *MYCN*-driven neuroblastoma (122, 123). MLN8237 destabilizes N-MYC and synergizes with BCL2/BCLxL inhibitor (venetoclax or navitoclax) to kill *MYCN*-amplified tumor cells including neuroblastoma and rhabdomyosarcoma (124, 125). Since the degradation of N-MYC is regulated in part by a kinase-independent function of Aurora-A, CD532, a conformation-disrupting inhibitor of Aurora-A, acts as a more potent N-MYC inhibitor than the kinase activity inhibitor MLN8237 in neuroblastoma (126).

Polo-like kinase 1 (PLK1), a serine/threonine kinase that promotes G2/M-phase transformation, has an elevated expression level in high-risk neuroblastoma and is associated with poor prognosis of patients (127). PLK1 interacts with and phosphorylates FBXW7, promoting auto polyubiquitination and proteasomal degradation of FBXW7, which counteracts FBXW7-mediated degradation of N-MYC (128). In turn, stabilized N-MYC directly enhances the transcription of *PLK1*, forming a positive feedforward regulatory loop that reinforces the progress of *MYCN*-driven cancers. Inhibitors of *PLK1*, such as BI6727 and BI2356, preferentially trigger apoptosis of *MYCN*-amplified neuroblastoma and small cell lung cancer, and this therapeutic efficacy is synergistically enhanced by combined use with antagonists of anti-apoptotic B cell lymphoma 2 (BCL2) (128). UME103 and 9b, two novel dual PLK1 and BRD4 inhibitors, show better antitumor activity by inhibiting the transcription of *MYCN* gene and promoting the degradation of N-MYC protein (129, 130).

Ubiquitin-specific protease 7 (USP7) regulates the stability and activity of N-MYC in neuroblastoma (131). USP7 directly binds to N-MYC, deubiquitinates it, which preventing degradation of N-MYC by the 26S proteasome. The expression of USP7 is enhanced in patients of neuroblastoma with poorer prognosis. A small molecular inhibitor of USP’s deubiquitinase activity, P22077, destabilizes N-MYC, thereby markedly repressing the growth of *MYCN*-amplified human neuroblastoma cell lines in xenograft mouse models (131). Novel, selective inhibitors of USP7, USP7-055, and USP7-797, have been developed recently for tumor therapy including *MYCN*-amplified neuroblastoma (132).

## *NCYM*, a cis-Antisense Gene of *MYCN*

An interesting feature of *MYCN* gene is its cis-antisense transcript called *NCYM*. NCYM was initially recognized as a large non-coding RNA (133, 134), while recent studies indicate it encodes a *de novo* evolved protein that promotes tumor progression (135). The transcription of *NCYM* begins from intron 1 of the *MYCN* gene in the opposite direction to that of the *MYCN*, ultimately generating NCYM protein with 109 amino acids (**Figure 2**) (135). As a cis-antisense gene of *MYCN*, *NCYM* is always co-amplified with *MYCN* (136). Both coding and noncoding transcripts of *NCYM* contribute to higher N-MYC expression. NCYM stabilizes N-MYC protein by inhibiting the activity of GSK3β, thereby preventing phosphodegron-mediated N-MYC degradation (135). Noncoding transcript variants of *NCYM* may reinforce *MYCN* translation *via* expelling exon 1b through alternative splicing or promoter shift (136). MYCN stimulates transcription of both *NCYM* and *MYCN*, forming a positive regulatory loop and leading to high expression of both genes (137).

NVP-BEZ235, a dual inhibitor of both phosphoinositide 3-kinase (PI3K) and mTOR, promotes the degradation of N-MYC by GSK3β activation and effectively decreases tumor burden in the *MYCN* transgenic mouse. In contrast, NVP-BEZ235 cannot prolong the survival of the *MYCN*/*NCYM* double transgenic mice (135). This might be related to the N-MYC-independent functions of NCYM, *e.g.*, NCYM-mediated inhibition of GSK3β also lead to the stabilization of β-catenin, which promotes bladder cancer progression (138); NCYM promotes generation of MYC-nicks, cytoplasmic cleavage products of N-MYC and C-MYC, which inhibits apoptosis and enhances cancer cell migration (139). TAp63, an isoform of *p63* protein and a *p53* family protein, suppresses *MYCN*/*NCYM* bidirectional transcription, repressing neuroblastoma growth (140). Thus, the implication of *NCYM* gene in *MYCN*-driven tumors increases complexity and contributes to treatment resistance.

## Somatic Mutation of *MYCN*

In addition to deregulated expression of N-MYC due to gene amplification or dysregulation at mRNA and protein levels, a recurrent somatic mutation, proline 44 to leucine (P44L) (**Figure 1A**), is identified in various tumors (141), including, glioma (142), neoplastic cysts of the pancreas (143), medulloblastoma (144), neuroblastoma (145), Wilms tumor (67), skin basal cell carcinoma (146), T-lineage acute lymphoblastic leukemia (147), NUT midline carcinoma (148), Ovarian mesonephric-like adenocarcinoma (149). Notably, P44L mutation of N-MYC has occurred in 1.7% of high-risk neuroblastoma without *MYCN* amplification (145). Since the frequent occurrence of P44L switch in different cancers, this mutation has long been assumed as an activating one, but it has not been functionally or biochemically characterized until recently (28). KE Mengwasser compared the function of P44L mutant with the wild type N-MYC in terms of promoting proliferation, and they found that P44L N-MYC mutant displayed 2- and 4.5-fold higher log2-fold-change in pancreas cells and breast cells, respectively (150). Similarly, Liu et al. observed a modest but significantly shorter latency for the induction of highly penetrant T-lineage leukemia in P44L N-MYC expressing cells than that of wild-type N-MYC expressing cells (147). These evidences solidly confirm that P44L N-MYC is indeed an activating mutation.

Mechanistically, as P44L mutation site locates adjacent to the conserved phosphor-degron sites recognized by E3 ubiquitin ligases FBXW7 and HUWE1 (**Figure 1A**), a hypothesis was proposed in which P44L mutation could perturb the interaction between these ligases and N-MYC substrate, therefore, prevented N-MYC degradation and enhanced oncogenicity (147). Consistently, Liu et al. show that the degradation of the N-MYC protein is significantly delayed in the P44L mutated type than that of the wild type after the cells are treated with cycloheximide to block protein translation (147). However, Bonilla et al. display that the interacting with FBXW7 is not affected by the P44L mutation, instead, the autoubiquitination of FBXW7 is increased in the presence of P44L mutation, suggesting a different mechanism for the enhanced stability of P44L N-MYC (146). Furthermore, the P44L mutation is associated with increased mRNA levels of *MYCN* in neuroblastoma (145). A previous study shows that MYCN can be directly recruited to the intron1 region of its own gene which contains two putative E-box sites and thus promotes its own transcription in neuroblastoma cells (151). Considering this positive auto-regulatory loop, it is possible that P44L mutation enhances *MYCN* mRNA level through the auto-activating mechanism with the more stable form of N-MYC protein.

## Synthetic Lethal Interaction With Deregulated *MYCN*

The concept of synthetic lethality means targeting specific targets including proteins and metabolites that are essential for the viability of tumor cells with specific physiology, such as N-MYC overexpression. This strategy can kill cancer cells only while spares normal counterpart. For instance, checkpoint kinase 1 (CHK1) is a key player in the DNA damage checkpoint control, and inhibition of CHK1 sensitizes cells to additional genomic instability (152). Overexpression of N-MYC causes replication stress and DNA damage by the ectopic replication-fork firing, which results in remarkably higher sensitivity of N-MYC overexpressing tumors to CHK1 inhibition, and thereby CKH1 inhibition is synthetic lethal with N-MYC overexpression (153, 154). Similarly, we demonstrate that N-MYC sensitizes neuroblastoma cells to apoptosis induced by various death ligand or DNA-damaging drugs (155, 156). These results indicate targeting DNA repair system or drugs causing DNA damage could be synthetic lethal in *MYCN*-driven tumors. Recent studies reveal various strategies based on N-MYC-mediated synthetic lethality, including glutaminase inhibition or glutamine deprivation (157), BCL2 inhibition (125), eliminating SKP2 complexes (158), kinesin spindle protein (KSP) inhibition (159), G9a inhibition (160), poly (ADP-ribose) polymerase (PARP) inhibition (161, 162).

## Conclusion and Perspectives

Here we describe the regulatory network of *MYCN* expression (**Figure 2**). Multiple mechanisms can cause abnormal level of N-MYC, including gene amplification, enhanced transcription, translation and protein stability. Various therapeutic targets have been found to address N-MYC overexpression based on knowledge of these regulatory mechanisms. However, strategies that globally inhibiting gene expression (such as inhibiting CDK7 and BDR4) has not yet convincingly demonstrated that these inhibitors specifically target tumors with high N-MYC level, nor have these inhibitors reached advanced stages in clinical trials (16). Although directly and specifically targeting N-MYC has not yet been available, promise remains in developing new approaches to effectively treat *MYCN*-driven tumors. For examples, short interfering RNA (siRNA)-mediated silence of *MYCN* induces neurogenesis and inhibits proliferation in neuroblastoma models resistant to retinoic acid (163). Clinical applications of siRNA are developing and the first siRNA-based drug Patisiran (Onpattro) was approved for clinical use to treat transthyretin amyloidosis by the U.S. Food and Drug Administration (FDA) in 2018 (164). In addition, Yoda et al. identify a pyrrole-imidazole polyamide, MYCN-A3, able to directly target *MYCN* amplicons, which specifically reduces copy number and suppresses gene expression of *MYCN* (165).

## Author Contributions

RL wrote the manuscript. PS drew the cartoon figures. ZW and CY collected the articles. HC provided the idea and revised the manuscript. All authors contributed to the article and approved the submitted version.

## Funding

This research was supported by the National Key Research and Development Program of China (2016YFC1302204, 2017YFC1308601), the National Natural Science Foundation of China (81872071, 81672502), the Natural Science Foundation of Chongqing (cstc2019jcyj-zdxmX0033), and Chongqing University Innovation Team Building Program funded projects (CXTDX201601010).

## Conflict of Interest

The authors declare that the research was conducted in the absence of any commercial or financial relationships that could be construed as a potential conflict of interest.
